# Influence of Semiconductor Morphology on Photocatalytic Activity of Plasmonic Photocatalysts: Titanate Nanowires and Octahedral Anatase Nanoparticles

**DOI:** 10.3390/nano9101447

**Published:** 2019-10-11

**Authors:** Zhishun Wei, Maya Endo-Kimura, Kunlei Wang, Christophe Colbeau-Justin, Ewa Kowalska

**Affiliations:** 1 Hubei Provincial Key Laboratory of Green Materials for Light Industry, Hubei University of Technology, Wuhan 430068, China; 2 Institute for Catalysis (ICAT), Hokkaido University, N21 W10, Sapporo 001-0021, Japan; m_endo@cat.hokudai.ac.jp (M.E.-K.); kunlei@cat.hokudai.ac.jp (K.W.); 3 Laboratory of Physical Chemistry, CNRS UMR 8000, Université Paris-Saclay, 91400 Orsay, France; christophe.colbeau-justin@u-psud.fr

**Keywords:** noble metal, faceted titania, octahedral particle, titanate nanowire, plasmonic photocatalysis, photocatalyst morphology, morphology-governed activity, gold, silver, platinum

## Abstract

Octahedral anatase particles (OAP) with eight exposed and thermodynamically most stable (101) facets were prepared by an ultrasonication-hydrothermal (US-HT) reaction from potassium titanate nanowires (TNW). The precursor (TNW) and the product (OAP) of US-HT reaction were modified with nanoparticles of noble metals (Au, Ag or Pt) by photodeposition. Samples were characterized by X-ray diffraction (XRD), X-ray photoelectron spectroscopy (XPS), diffuse reflectance spectroscopy (DRS), scanning transmission electron microscopy (STEM) and time-resolved microwave conductivity (TRMC). The photocatalytic activity was investigated in three reaction systems, i.e., anaerobic dehydrogenation of methanol and oxidative decomposition of acetic acid under UV/vis irradiation, and oxidation of 2-propanol under vis irradiation. It was found that hydrogen liberation correlated with work function of metals, and thus the most active were platinum-modified samples. Photocatalytic activities of bare and modified OAP samples were much higher than those of TNW samples, probably due to anatase presence, higher crystallinity and electron mobility in faceted NPs. Interestingly, noble metals showed different influence on the activity depending on the semiconductor support, i.e., gold-modified TNW and platinum-modified OAP exhibited the highest activity for acetic acid decomposition, whereas silver- and gold-modified samples were the most active under vis irradiation, respectively. It is proposed that the form of noble metal (metallic vs. oxidized) as well as the morphology (well-organized vs. uncontrolled) have a critical effect on the overall photocatalytic performance. TRMC analysis confirmed that fast electron transfer to noble metal is a key factor for UV activity. It is proposed that the efficiency of plasmonic photocatalysis (under vis irradiation) depends on the oxidation form of metal (zero-valent preferable), photoabsorption properties (broad localized surface plasmon resonance (LSPR)), kind of metal (silver) and counteraction of “hot” electrons back transfer to noble metal NPs (by controlled morphology and high crystallinity).

## 1. Introduction

Titanium(IV) oxide (titania, TiO_2_) has been widely investigated for environmental and energy applications due to many advantages, such as good stability, strong redox ability, nontoxicity (except nanomaterial toxicity), low cost and high availability [1,2,3]. It should be pointed that also other semiconductors have been used successfully for environmental applications, such as ZnO [4], WO_3_ [5], Cu_2_O [6], graphitic carbon nitride (g-C_3_N_4_) [7], SrTiO_3_ [8], NaTaO_3_ [9], KNbO_3_ [10], Ta_3_N_5_ [11] and potassium titanate (K_2_Ti_4_O_9_ [12], K_2_Ti_6_O_13_ [13], K_2_Ti_8_O_17_ [14], K_2_TiO_3_ and K_3_Ti_8_O_17_ [15]). Moreover, some of them have shown higher activity than that by titania even under UV irradiation, e.g., ZnO for degradation of terephthalic acid [16], 2-phenylphenol [17], dyes (e.g., Reactive Blue 19 [18]) and benzoic acid (but not formic acid) [19]. However, it should be reminded that on the contrary to titania, ZnO is not stable at acidic conditions [17,20,21]. Additionally, usually only two photocatalysts have been compared (one TiO_2_ and one ZnO), which does not guarantee that other ZnO materials with different properties would have also higher activity than titania samples, as confirmed by huge differences in activities of 35 titania photocatalysts [22].

A lot of studies have already indicated the influence of semiconductor structural/physical properties on the photocatalytic activity, and it is generally considered that higher crystallinity, larger specific surface area, smaller crystallite size and specific morphology (exposed crystal facets) result in higher photocatalytic activity [22,23,24,25,26]. For example, it has been proposed that octahedral anatase particles (OAPs) has high photocatalytic activity because of the preferential distribution of shallow than deep electron traps (ETs), which could efficiently delay or prevent the recombination of electron-hole pairs [27]. Different methods of titania synthesis have been reported, including hydrothermal reaction (HT), solvothermal, sol-gel, electrosynthesis, soft- and hard-templating, micelle and inverse micelle, direct oxidation, chemical and physical vapor deposition, sonochemical, microwave, gas-phase and green synthesis (“bio-reduction” using different biological extracts) [26,28,29,30,31]. Probably, the HT method is one of the most popular for titania (and other nanomaterials) synthesis (as also confirmed by special issues on hydrothermal synthesis, e.g., in J. Nanomater. “Hydrothermal Synthesis of Nanomaterials”, 2019 and Materials “Conventional and Microwave Hydrothermal Synthesis of Functional Materials”, 2019/2020), due to feasible preparation of required nanostructures by simple changing of process conditions [23]. Advantages and disadvantages of various methods, including reproducibility, process duration, purity, costs, safety, etc., have been discussed previously, and might be found in the following papers [31,32,33,34,35]. Although particulate titania is one of the most frequently studied (and even commercially used), other nanostructures are also intensively investigated, such as nanotubes, nanocoils, nanoflowers and nanoplates. In particular, 1-D nanostructures (nanotubes, nanowires, nanorods, nanoribbons and nanobelts) have been proposed as a good candidate for photocatalysis, due to enhanced charge carriers’ separation [36,37,38]. Therefore, in this study, the comparison between zero-dimensional (0D) and one-dimensional (1D) semiconductors, i.e., faceted anatase particles vs. titanate nanowires, has been investigated.

Even though titania is one of the most active photocatalysts, the charge carriers’ recombination (typical for all semiconductors) causes much lower quantum yields of photocatalytic reactions than expected 100%. Moreover, broad bandgap of titania (ca. 3.0–3.2 eV; depending on crystalline form) results in poor overlap of its photoabsorption with the solar spectrum (absorption edge at ca. 385–410 nm), and thus the activity under natural solar radiation (consisting of only ca. 3%–4% of UV) is usually very low. Accordingly, many methods of titania modification have been proposed to improve its photocatalytic performance, e.g., doping [39,40,41], surface modification [42,43,44] and coupling with other materials (heterojunctions [6,45,46,47,48]). Some reviews on titania modification and doping discussing advantages and disadvantages of various methods might be found here [39,49,50,51,52,53,54,55]. Moreover, preparation of highly crystalline polyhedral TiO_2_ particles with crystal facets in definite orientations has been proposed as promising strategy to retard charge carriers’ recombination [56,57,58]. To obtain visible light-responsive photocatalysts, various compounds have been used as surface modifiers of titania, e.g., metallic deposits [44] metal complexes [59] and non-metals (adsorbed anions [60] or chemical compounds [61]). Among them, plasmonic nanoparticles (NPs), i.e., NPs of noble metals (NMs) with localized surface plasmon resonance (LSPR) at vis range of solar spectrum, have attracted increasing attentions as promising modifiers, and such materials (semiconductors with plasmonic deposits) are presently known as plasmonic photocatalysts [55,62,63,64,65].

To obtain highly active plasmonic photocatalysts and to clarify the key-factors of photocatalytic activity under vis irradiation, two types of semiconductors have been chosen for present study, i.e., octahedral anatase particles (OAPs) and potassium titanate nanowires (TNWs; precursor of OAPs). To the best of our knowledge, the comparison of photocatalytic activity of titania and potassium titanate has not been reported yet. In this study, NM-modified OAPs (prepared and already reported previously [66], except Pt-modified OAPs) and TNWs were comparatively analyzed to find how the different types of metals (Au, Ag and Pt) and their supports could influence the resultant properties, and thus photocatalytic activities in different reaction systems.

## 2. Materials and Methods

### 2.1. Preparation of Photocatalysts

Potassium titanate nanowires (TNWs) were prepared by company (Earthclean Tohoku Co. Ltd., Sendai, Miyagi, Japan) from titania P25 (commercial titania sample containing anatase (76%–80%), rutile (13%–15%) and amorphous titania (6%–11%) [67]) by HT reaction in a 17 mol L^−1^ potassium hydroxide solution for 20 h at 383 K (named as TNW) [68]. Octahedral anatase particles (OAPs) with eight exposed thermodynamically stable (101) facets were prepared by an ultrasonication-hydrothermal (US-HT) reaction [27]. TNWs (267 mg) were ultrasonically dispersed in Milli-Q water (40 mL) for 1 h at room temperature, and thus-prepared suspension was poured in a sealed Teflon bottle (100 mL), to which additional 40 mL of Milli-Q water was added. The bottle was heated for 6 h at 433 K in an oven without agitation. After cooling, the suspension was centrifuged, and the sedimented titania was washed with water and dried under vacuum (353 K, 12 h). The thus-obtained OAP-containing samples (named as OAP) were used for further studies.

NM (gold, silver or platinum; 2 wt% in respect to semiconductor) was photodeposited on TNW, OAP and commercial titania samples in a sealed and deaerated (15-min argon pre-bubbling) pyrex tube containing semiconductor (500 mg), methanol as a hole scavenger (25 mL, 50 vol%) and aqueous solution of NM precursor (HAuCl_4_·4H_2_O, AgNO_3_ and H_2_PtCl_6_·6H_2_O, respectively) under UV/vis irradiation. Details of photodeposition method were presented previously [69]. In brief, under UV irradiation the photogenerated electrons reduce metal cations, and thus obtained metallic nanocluster/nanoparticles (NPs) are in situ deposited on the semiconductor surface. This method is very efficient, due to the direct electronic contact between semiconductor and NM NPs, since metal cations are reduced by photogenerated electrons directly on the semiconductor surface. Accordingly, the linear evolution of hydrogen (methanol dehydrogenation during photodeposition) indicates that the reduction reaction is complete and all metal cations have been reduced and their NPs are deposited on the support (as confirmed by atomic absorption spectroscopy previously [70]).

The codes of samples were defined as TNW (potassium titanate nanowires), OAP (anatase sample composed mainly of octahedral anatase particles), FP-6, ST01 and TIO10 (three commercial anatase titania samples with properties shown in Table S1); Au/TNW, Ag/TNW and Pt/TNW (TNW sample with deposited NPs of gold, silver and platinum, respectively); Au/OAP, Ag/OAP and Pt/OAP (OAP sample with deposited NPs of gold, silver and platinum, respectively). Analogically, commercial titania samples modified with noble metals were named as Au/FP-6, Ag/FP-6, Pt/FP-6, Au/ST01, Ag/ST01, Pt/ST01, Au/TIO10, Ag/TIO10 and Pt/TIO10. HAuCl_4_·4H_2_O, AgNO_3_ and H_2_PtCl_6_·6H_2_O are supplied from Wako (Wako Pure Chemical industries, LTD. Osaka, Japan), ST01 (Ishihara Sangyo Kaisha, LTD. Tokyo, Japan); FP-6 (Showa Titanium, LTD. Nara, Japan) and TiO10 (Titan Kogyo, LTD. Tokyo, Japan).

### 2.2. Samples’ Characterization

Photoabsorption properties were analyzed by diffuse reflectance spectroscopy (DRS; JASCO V-670 equipped with a PIN-757 integrating sphere, JASCO, LTD., Pfungstadt, Germany). Barium sulfate or bare semiconductor samples (TNW or OAP) were used as references for DRS analysis. Crystalline properties of photocatalysts (crystalline phase content, crystallite size) were determined by X-ray powder diffraction (XRD; Rigaku intelligent XRD SmartLab with a Cu target, Rigaku, LTD., Tokyo, Japan). Crystallite size of anatase was estimated from the corrected width of (101) anatase diffraction peak using the Scherrer equation.

Chemical composition of the surface (chemical state and content of elements) was analyzed by X-ray photoelectron spectroscopy (XPS; JEOL JPC-9010MC with MgKα X-ray, JEOL, LTD., Tokyo, Japan). The morphology of samples was investigated by high resolution transmission electron microscopy (HR-TEM, JEOL JEM-2100F, JEOL LTD., Tokyo, Japan) and scanning transmission electron microscopy (STEM), equipped with an energy-dispersive X-ray spectroscopy (STEM-EDS, HITACHI HD-2000, Hitachi LTD., Tokyo, Japan). Summarized characteristics of bare OAP and TNW samples are presented in Table S1.

Charge-carrier dynamics was estimated by time-resolved microwave conductivity (TRMC) method. The incident microwave of 36.8 GHz was generated by a Gunn diode of the *K*α band, and UV laser pulses were obtained by the third harmonic of a 1064 nm Nd:YAG laser (10 Hz) with full width at half maximum of ca. 10 ns. Details of measurement and data processing have been reported elsewhere [9,16].

The photocatalytic activity of prepared samples was evaluated in three reaction systems: (1) Decomposition of acetic acid under UV/vis irradiation, (2) dehydrogenation of methanol under UV/vis irradiation and (3) oxidation of 2-propanol under vis irradiation (λ > 420 nm: Xe lamp, water IR filter, cold mirror and cut-off filter Y45). For activity tests, 50 mg of photocatalyst was suspended in 5 mL of aqueous solution of (1) methanol (50 vol%), (2) acetic acid (5 vol%) and (3) 2-propanol (5 vol%). The 35-mL testing tubes were sealed with rubber septa, continuously stirred and irradiated in a thermostated water bath. Amounts of liberated (1) carbon dioxide in gas phase, (2) hydrogen in gas phase and (3) acetone in liquid phase (after powder separation) were determined by gas chromatography (gas chromatography with thermal conductivity detector, GC-TCD, Shimadzu, Corp. Tokyo, Japan (1–2) and gas chromatography with flame ionization detector, GC-FID, Shimadzu, Corp. Tokyo, Japan (3)), details and experimental set-ups have already been described elsewhere [69,71].

## 3. Results

### 3.1. Photodeposition of Noble Metals

**Figure 1:**
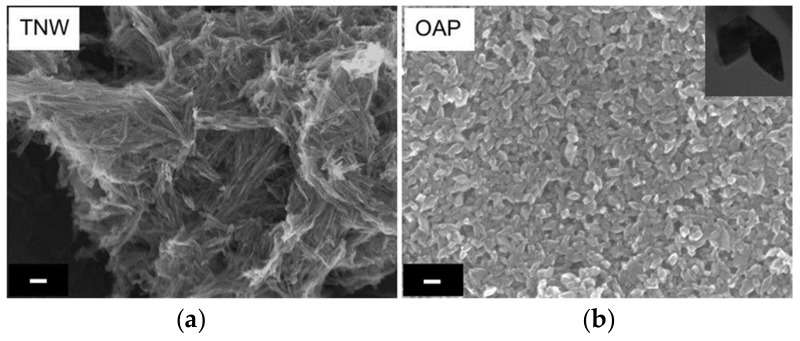
SEM images of: (**a**) Potassium titanate nanowire (TNW) and (**b**) octahedral anatase particle (OAP). Scale bars correspond to 50 nm.

**Figure 2:**
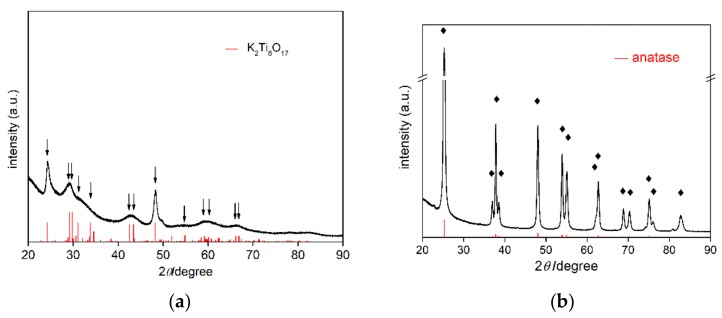
XRD patterns of: (**a**) Bare TNW, and (**b**) bare OAP, with marked K_2_Ti_8_O_17_ and anatase peaks, respectively (red patterns from PDF cards No 9874 and No 8214, respectively).

SEM images of bare TNW and OAP are shown in Figure 1. The obtained OAP particles were smaller than 30 nm, whereas TNW were thinner than 10 nm and longer than 100 nm. Therefore, specific surface area of TNW was almost three times larger than that of OAP reaching ca. 360 m^2^ g^−1^ (124 m^2^ g^−1^ for OAP). XRD patterns of bare TNW and OAP are shown in Figure 2. The properties of bare TNW, OAP and commercial titania samples (used here for comparison) are summarized in Table S1. TNW were mainly composed of K_2_Ti_8_O_17_, whereas anatase was the major phase in other samples of crystallite sizes in the range from 8 to 15 nm. Only TNW had wire-like morphology (1D), other samples consisted of fine NPs (0D), including OAP NPs with well-defined shape of octahedron.

All samples (TNW, OAP and commercial titania) were modified with NM (Au, Ag or Pt) NPs by photodeposition method. 2 wt% of NM (in respect to semiconductor) has been selected for this study since usually it results in one of the highest levels of activity under both UV and vis irradiation for various NMs and in different reaction systems, and this content is sufficient for reliable characterization of samples (XRD/XPS) [67,70,72,73]. Moreover, in the present study the optimal content of gold has been investigated for TNW in two reaction systems, as shown in Figure S3. Indeed, it was confirmed that 2 wt% resulted in the highest photocatalytic activity under both UV/vis irradiation (H_2_ evolution) and vis irradiation (acetone generation). A decrease in the activity for larger than 2 wt% content is not surprising (and commonly reported), due to the shielding effect [70,74], as clearly shown in Figures S1 and S2, i.e., the color of gold-modified TNW become darker with an increase in gold content from 0.01 wt% to 5 wt%.

**Figure 3:**
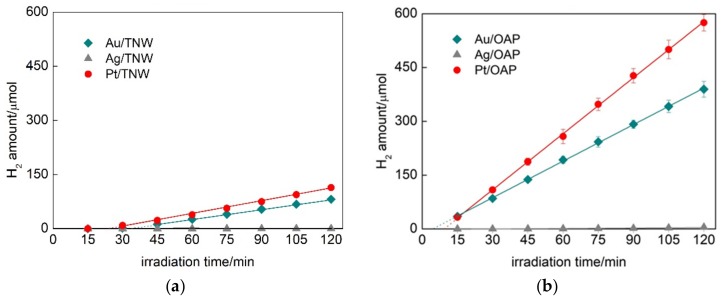
Hydrogen evolution during metal photodeposition on: (**a**) TNW and (**b**) OAP.

During NM photodeposition the color of suspension changed from white to violet, brown and grey for Au, Ag and Pt, respectively, confirming that NPs of zero-valent metal were deposited on the surface of semiconductors. The photodeposition course showing rates of hydrogen evolution and induction periods (during which metal cations were reduced and zero-valent NPs were formed) are shown in Figure 3 and Figure S10. It was found that deposition of platinum and gold was much faster than that of silver, and resulted in the highest rates of hydrogen evolution independently on the semiconductor kind. The induction periods for silver-modified samples were much longer than that for gold- and platinum-ones reaching more than one hour to complete metal cations’ reduction (observed by linear hydrogen evolution).

The metal-modified OAP samples showed much higher (3–4×) photocatalytic activity for hydrogen evolution than metal-modified TNW samples. For example, the hydrogen evolution rates were 3.4, 5.2, 0.9 and 1.2 µmol min^−1^ for Au/OAP, Pt/OAP, Au/TNW and Pt/TNW, respectively. The induction periods (intersection with the x-axis in Figure 3) during metal deposition on OAP samples (4.6 min for Au/OAP and 9.4 min for Pt/OAP) were much shorter than those for the TNW samples (33.6 min for Au/TNW and 23.8 min for Pt/TNW). Both higher activity (reaction rates) and shorter induction periods for OAP samples than those for TNW samples suggest faster separation of charge carriers on OAP, probably due to high crystallinity, anatase presence and/or octahedral morphology. The longest induction periods (>60 min) as well as the lowest rates of hydrogen evolution were observed for silver-modified samples due to the lowest work function (4.14–4.46 eV) [75]. In the case of metal deposition on commercial titania samples (Figure S10), similar results were found, i.e., the platinum and silver deposition was the fastest and the slowest, respectively, with the highest and the lowest rates of hydrogen generation.

### 3.2. Characterization of Samples

Although, metal modification of titania resulted in coloration of all samples due to deposition of zero-valent NMs, only gold and platinum samples kept the same color after photodeposition (violet and grey, respectively). In contrast, the color of silver samples changed to light brown-violet, since silver (as less noble) was easily oxidized under aerobic conditions, and thus resultant photocatalysts possessed both metallic and oxidized form of silver (e.g., zero-valent core and oxide shell [76]).

**Figure 4:**
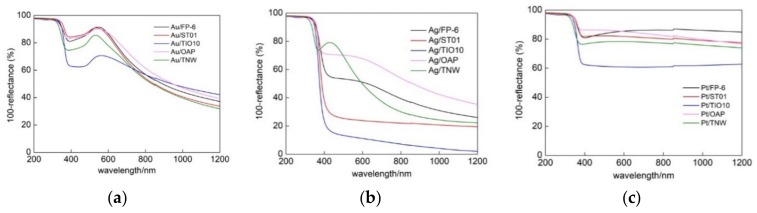
Diffuse reflectance spectroscopy (DRS) spectra of noble metal (NM)-modified samples (TNW, OAP and commercial titania: FP-6, ST01 and TiO10) for: (**a**) Au, (**b**) Ag and (**c**) Pt; taken with BaSO_4_ as a reference.

DRS spectra of all samples (FP-6, ST01, TIO10, OAP and TNW) modified with different metals (Au, Ag and Pt) are shown in Figure 4 and Figures S4 and S5. The absorption peak positions (LSPR) were at ca. 410–420 nm for silver- and platinum-modified samples and at ca. 530–560 nm for gold-modified samples, corresponding well with the literature data for spherical NM NPs of 5–30 nm [48,49,50,51,52,53]. The maximum of LSPR slightly differed between samples, e.g., 530, 534, 545, 546 and 561 nm for gold-modified TNW, ST01, FP-6, OAP and TIO10, respectively, suggesting that the smallest gold NPs were deposited on TNW, whereas the largest on TIO10. Indeed, analysis of crystallite sizes (XRD) confirmed that the smallest crystallites of 5.4-nm size were deposited on TNW, and the largest ones (10 nm) on TiO10. It should be pointed that the properties of photodeposited metals depend directly on the properties of the support. For example, in the case of 15 commercial titania samples, an increase in the crystallite size of titania resulted in an increase in the size of gold NPs [40]. It was suggested that crystalline defects were mainly responsible for this dependence since the content of defects (electron traps, ETs) was indirectly proportional to crystalline/particle sizes (Specific surface area correlates with the content of ETs [54]), and as was reported previously, gold NPs were mainly formed on these defects (nucleation sites) [55]. Similarly, in this study, larger gold NPs were deposited on titania with larger crystallites, except faceted titania (OAP) with the largest crystallites of titania (17 nm) and one of the smallest crystallites of gold (5.7 nm). It should be pointed that the content of ETs in OAP is much lower than in commercial titania samples of similar specific surface area [9,56]. Therefore, it is proposed that defects in faceted NPs (with well-defined morphology) differed from that in commercial titania samples. Similarly, defect-dependent activity was suggested for OAP samples with almost the same surface properties (crystallite size, crystallinity and specific surface area) and different morphology (content of faceted NPs) [9]. It was proposed that the content of shallow ETs was larger in faceted NPs (almost same content of total ETs in various OAP samples, but much larger electron mobility in the samples with better morphology (larger content of faceted NPs), as suggested from photoacoustic spectroscopy (PAS) and TRMC, respectively).

In the case of platinum and silver deposition, the LSPR peaks overlapped with titania/titanate peaks, and thus DRS spectra with bare semiconductors (respective titania sample and TNW) as references were also taken, and are shown in SI (Figures S4 and S5). Although, for all modified samples the clear absorption peaks at visible range were observed (confirming that respective metals were successfully deposited on titania), only in the case of gold, the maximum of vis absorption could clearly correlate with LSPR (at ca. 530–561 nm). In contrast, photoabsorption spectra of silver-modified samples were very broad, in which two maxima could be distinguished, e.g., at ca. 410 and 520 nm for Ag/ST01 and Ag/FP-6, and at 418 and ca. 600 nm for Ag/OAP. The absorption at shorter wavelengths was surely caused by LSPR of fine silver NPs, but photoabsorption at longer wavelengths could result from either the presence of very large and/or non-spherical NPs of silver (e.g., rod-like NPs possessing transverse and longitudinal LSPR at shorter and longer wavelengths, respectively) or co-existence of silver oxides with narrower bandgap than that of titania (ca. 2.2 eV [77]). The former could be neglected as fine NPs of silver were formed on titania, as shown by XRD and STEM analyses (discussed in the next parts). In the case of platinum deposition, the wavelengths at maximal absorption were difficult for evaluation since almost same absorption at whole vis range was obtained, as typical for grey/black samples, and already reported for platinized titania [78,79].

**Figure 5:**
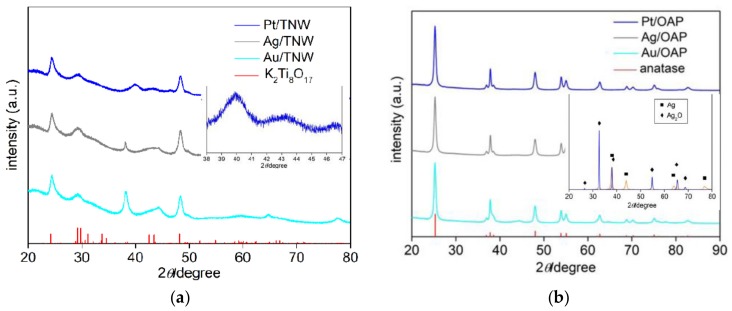
XRD patterns of NM-modified: (**a**) TNW and (**b**) OAP with marked K_2_Ti_8_O_17_ and anatase peaks, respectively (red patterns from PDF cards No 9874 and No 8214, respectively); (inserts) XRD patterns of: (**a**) Pt/TNW after subtraction of K_2_Ti_8_O_17_ peaks and (**b**) Ag/OAP after subtraction of anatase peaks with marked Ag (∎) and Ag_2_O (◆).

The surface modification with NM did not change crystalline properties of supports, as presented in Figure 5. The position of anatase and titanate peaks was not shifted, confirming the nature of surface modification rather than doping. The intensity of NM peaks was very low due to low NM content and fine crystallites. However, after subtraction of titanate (K_2_Ti_8_O_17_) and anatase peaks, clear peaks for NM could be observed, as shown in inserts in Figure 5 for exemplary patterns of NM-modified samples (Pt/TNW and Ag/OAP, respectively).

**Figure 6:**
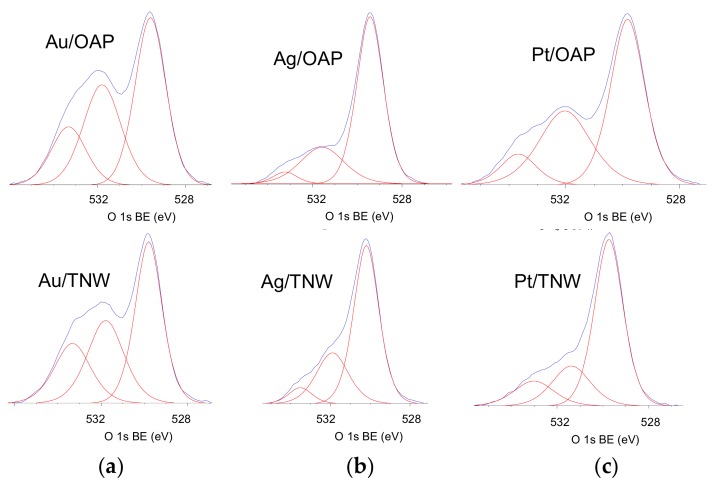
XPS results for O 1s for NM-modified samples: OAP (top) and TNW (bottom) for: (**a**) Au, (**b**) Ag and (**c**) Pt.

**Figure 7:**
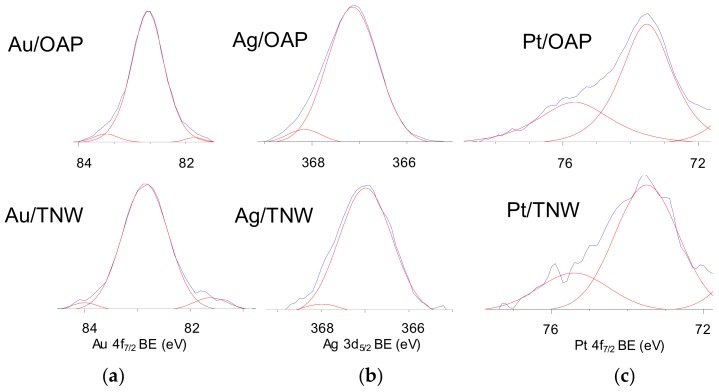
XPS results for Au 4f_7/2_, Ag 3d_5/2_ and Pt 4f_7/2_ for NM-modified samples: OAP (top) and TNW (bottom) for: (**a**) Au, (**b**) Ag and (**c**) Pt.

The surface chemical composition of metal-modified OAP and TNW samples was estimated by XPS analysis, and the data are shown in Table 1, Table 2 and Table 3 and Figure 6 and Figure 7. The binding energies for titanium, oxygen, carbon and NMs (gold, silver and platinum) were estimated after deconvolution in accordance to published reports on XPS analysis [80,81,82,83,84,85]. Narrow scan XPS spectra for O 1s, Au 4f_7/2_, Ag 3d_5/2_ and Pt 4f_7/2_ are shown in Figure 6 and Figure 7. It was found that the metal content on the surface depended on its kind and the support, e.g., the metal contents on the OAP were higher than those on the TNW (4.4 wt% v. 2.2 wt% for gold, 6.7 wt% v. 3.6 wt% for silver, 10.4 wt% v. 4.0 wt% for platinum), meanwhile, the highest and lowest content of metal was obtained for platinum- and gold-modified samples, respectively. Gold and platinum were predominantly in the zero-valent form both on OAP and TNW supports (Table 2). In contrast, silver existed mainly as Ag^+^ (93.7% for Ag/OAP and 94.3% for Ag/TNW), and minority of zero-valent silver was also detected (6.3% and 5.7%, respectively), confirming previous findings on co-existence of Ag/Ag^+^ (in the case of less noble metals, such as Ag and Cu [86,87]).

The ratio of oxygen to titanium (Table 1) exceeded stoichiometric one (2.0 for TiO_2_, 2.125 for K_2_Ti_8_O_17_) for OAP samples, reaching 5.2 for bare OAP and 2.1–3.33 for metal-modified OAP, which has already been reported for various titania samples. For example, the surfaces of titania samples prepared by microemulsion method, laser ablation, hydrothermal reaction and gas-phase method were highly enriched with oxygen (O/Ti = 4.6 [88], 2.5 [89], 2.1–5.0 [90] and 7.7 [66], respectively). The modification with metals resulted in a decrease in O/Ti ratio, suggesting that metals replaced surface oxygen, which was also confirmed by a decrease in the content of OH groups on the surface (Table 3). In contrast, the surface of bare TNW was slightly reduced since only 1.9 ratio of oxygen to titanium was obtained. Moreover, unlike NM-modified OAP, surface modification of TNW with NMs resulted in an increase in O/Ti ratio to 3.2, 2.4 and 2.3 for Au/TNW, Ag/TNW and Pt/TNW, respectively.

Deconvolution of oxygen peak into three peaks (Figure 6) suggests the presence of three forms of oxygen, i.e., (i) oxygen in the crystal lattice of TiO_2_ and K_2_Ti_8_O_17_ (in the case of OAP and TNW samples, respectively), (ii) C=O, Ti_2_O_3_ and OH groups bound to two titanium atoms and (iii) hydroxyl groups bound to titanium or carbon (Ti–OH and C–OH) at ca. 529.8 eV, 531.7 eV and 533.3 eV, respectively [85]. Deconvolution of carbon peak into three peaks indicates the presence of three forms of carbon, i.e., (i) C–C, (ii) C–OH and (iii) C=O at ca. 284.8 eV, 286.4 eV and 288.7 eV, respectively. The presence of carbon is typical for all titania samples (and other oxides), mainly due to carbon dioxide adsorption from surrounding air during sample preparation, which further forms bicarbonate and mono- and bidentate carbonate adsorbed on the titania/titanate surface [91]. The deconvolution results are summarized in Table 3. Considering chemical state of titanium, all samples consisted mainly Ti^4+^ with less than 5% of Ti^3+^. Interestingly, the modification with NMs resulted in an increase in the content of reduced form of titanium, which is reasonable considering that metals were photodeposited under reduced conditions (anaerobic; in the presence of hole scavenger). The platinum-modified samples had the lowest content of Ti^3+^ on the surface, i.e., 0.9% and 2.9% for Pt/OAP and Pt/TNW, respectively. In the case of OAP samples, the correlation between reducibility of NM cation and Ti^3+^ content was noticed, i.e., the higher NM reducibility was, the lower was the reduction of titanium (photogenerated electrons reduced fast and mainly NM cations, but not titanium).

**Figure 8:**
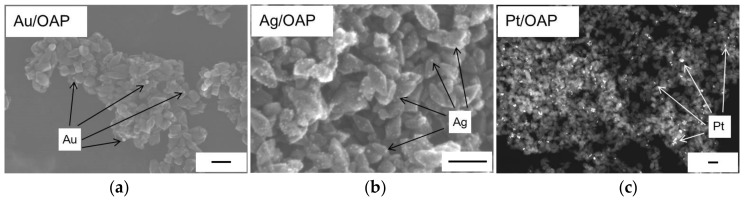
STEM images of: (**a**) Au/OAP (SE mode); (**b**) Ag/OAP (SE mode) and (**c**) Pt/OAP (ZC mode). Scale bars correspond to 50 nm.

Morphology of photocatalysts was studied by STEM, and exemplary images are shown in Figure 8. It was confirmed that NMs were deposited on the OAP surface successfully. Only metal-modified OAP samples were chosen for analysis due to much higher photocatalytic activity than that of TNW samples (as discussed further). It was found that NPs of platinum were the largest, whereas the smallest NPs were formed by silver, in contrary to crystalline sizes. It is proposed that speed of metal deposition determinates the particle size, i.e., fast photodeposition results in crystallites’ aggregation. Indeed, it was found that the slowing of metal photodeposition (e.g., initially aerobic conditions) resulted in a decrease in NPs’ size [92].

### 3.3. Photocatalytic Activity of Samples

**Figure 9:**
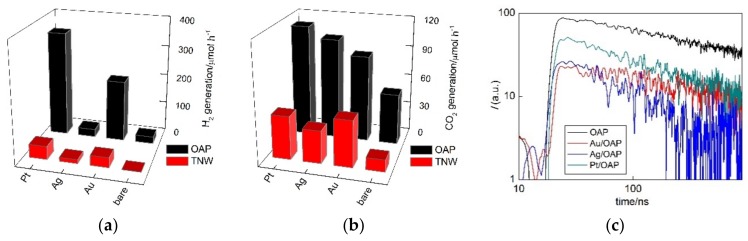
UV-vis-photocatalytic activity (**a**,**b**) of bare and NM-modified OAP and TNW samples for: (**a**) Methanol dehydrogenation and (**b**) decomposition of acetic acid; and (**c**) the time course of the time-resolved microwave conductivity (TRMC) signal for bare and NM-modified OAP samples after UV excitation (355 nm).

**Figure 10:**
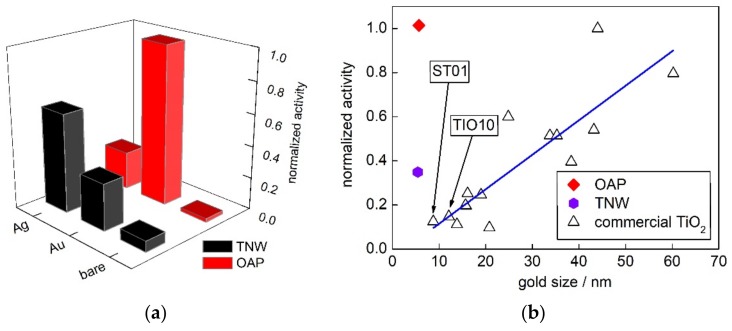
Vis-photocatalytic activity of bare and NM-modified samples: (**a**) Comparison of vis-activity of TNW and OAP samples; and (**b**) correlation between gold crystallite size and photocatalytic activity of Au-modified TNW, OAP and 15 commercial titania samples.

Photocatalytic properties were examined in three reaction systems, i.e., methanol dehydrogenation under UV/vis irradiation (H_2_ evolution; Figure 9a), oxidative decomposition of acetic acid under UV/vis (CO_2_ evolution; Figure 9b) and oxidation of propanol to acetone under vis irradiation (Figure 10).

It should be pointed that H_2_ evolution was also examined during sample preparation (Figure 3). However, sample drying could result in change of the properties (higher content of oxidized form of NM), and thus photocatalytic activity. For example, much different activities were obtained for Cu-modified faceted titania (OAP and decahedral anatase particles (DAP)) during in situ H_2_ evolution (photodeposition) and after sample drying, where it was proposed that formed heterojunctions between titania and copper oxides (Cu_2_O/TiO_2_ and CuO/TiO_2_) could be responsible for activity enhancement [66]. Here, for modification with Pt, Au and Ag, similar activities were achieved to those during metal deposition, suggesting that sample drying did not change the properties significantly. It is proposed that although Ag was mainly positive after drying, the reductive conditions during activity testing (exactly same as that during photodeposition) resulted in its renewed reduction (also observed by color change to darkener brown), and thus almost same activity as during sample preparation was obtained. For comparison, the activities of bare semiconductors were also tested, and the data obtained are shown in Figure 8a,b).

Although bare titania samples are usually inactive for hydrogen evolution, bare OAP possessed little activity, probably due to the presence of shallow ETs with high energy (detected by reversed double-beam photoacoustic spectroscopy, RDB-PAS [93]). Moreover, OAP activity exceeded highly (ca. 60 times) that by TNW (Figure 8 and Figure S6). The highest photocatalytic activities of platinum-modified samples agreed with the highest work function of Pt (5.7 eV vs. 5.1 eV (Au) and 4.3 eV (Ag)) [94,95,96], and thus formed Schottky barriers resulting in hindering of charge carriers’ recombination, as well as with the smallest overvoltage for hydrogen evolutions. It should be pointed that OAP samples after modification with NM showed much higher photocatalytic activity than that of respective TNW samples, especially for Au and Pt (Au/OAP and Pt/OAP were ca. six and eight times more active than Au/TNW and Pt/TNW, respectively).

Similarly, during oxidative decomposition of acetic acid, all OAP samples showed much higher photocatalytic activity than respective TNW ones, despite much lower specific surface area. It is proposed that high photocatalytic activity of OAP samples might be caused by: (i) High content of shallow ETs responsible for rapid electron transfer (instead of deep ET with permanent electron trapping) [27], (ii) efficient adsorption and reduction of oxygen on (101) facet [97], and (iii) highly crystalline form (small content of defects). Photocatalytic activity increased significantly after titania modification with NM, which should work as an electron pool. However, they did not correlate with work function of NM, and thus with the formed Schottky barrier. It was found that Pt/OAP and Au/TNW were the most active, then Ag/OAP and Pt/TNW and the least active were Au/OAP and Ag/TNW. The higher activity of Ag/OAP than Au/OAP could be explained by co-participation of heterojunctions in the overall activity, i.e., the overall activity might be composed of the activity of titania with zero-valent silver (silver as an electron pool) and the activity of formed heterojunctions (Ag_2_O/TiO_2_ and/or Ag/Ag_2_O/TiO_2_, as suggested by XRD, XPS and DRS analyses).

The role of NM as a scavenger for photogenerated electrons was confirmed by time-resolved microwave conductivity (TRMC) method (Figure 9c), which might evaluate the mobility and lifetime of charge carriers. The TRMC signal (arbitrary) increased with a 355-nm laser pulse (ca. 10 ns), which excited the photocatalyst to generate charge carriers (electrons and holes), and then decayed after the laser pulse in ns to µs time scale [9]. The signal intensity correlated with the mobility and the number of charge carriers, i.e., an overall mobility of charge carriers. In the TRMC measurement, two parameters were evaluated, i.e., the maximum intensity of TRMC signal (I_max_) and the rate of signal decay, corresponding to an overall mobility of photoexcited electrons after laser excitation and recombination of electron-hole pairs, respectively. The rate of signal decay is important since it is a key factor to reflect the recombination speed of photogenerated electron-hole pairs. Indeed, a decrease in *I*_max_ after OAP modification with NM confirmed that NM scavenged photogenerated electrons, delaying or avoiding the recombination with holes. Although, the TRMC method is very convenient and useful, the analysis and discussion on the TRMC results was quite complex. For example, the lowest *I*_max_ and the slowest rate of signal decay for Au/OAP might suggest its highest activity among all samples, as also reported for other titania samples modified with NM NPs and NM complexes (the lowest *I*_max_ but also the fastest signal decay for the most active samples) [98]. Here, the smallest decrease in *I*_max_, but also the fastest signal decay was observed for the most active sample (Pt/OAP). Similar (fast) decay was also observed for highly active Ag/OAP. Therefore, it is suggested that in contrast to bare titania samples, where the slowest signal decay confirmed the highest activity (long lifetime of charge carriers) [27,66], fastest signal decay indicated the fast electron capture by NM, and thus high photocatalytic activity. Similar results have already been presented for P25 titania modified with: (i) Mono- and bimetallic NPs (Ni and Pd) and Ag nanoclusters, i.e., the highest *I*_max_ and the fastest signal decay for the most active samples [99,100], respectively.

The vis activity (λ > 420 nm) of TNW and OAP samples are shown in Figure 10a. All activities were normalized to that of Au/OAP (as a reference). Although titania and potassium titanate were inactive under vis irradiation, bare OAP and bare TNW showed slight activity, and activity of TNW was much higher than that by OAP (Figure 10). The vis activity of bare semiconductors might result from ETs localized inside bandgap (“intrinsic bandgap narrowing”). Therefore, it is not surprising that not well-crystallized TNW (with possibly large content of defects) possessed vis activity.

Au/OAP and Ag/TNW showed the highest vis response among all samples (Pt-modified samples were not considered since they possessed high “dark” activity.). The highest activity for Au/OAP suggests that more photons might be used by LSPR of gold than that for silver, as LSPR of silver overlapped with that of titania (Figures S4 and S5). However, for TNW the highest activity was obtained for Ag/TNW, but not for Au/TNW with much better photoabsorption properties (Figure S4), but it should be reminded that LSPR of silver was suggested as more photocatalytically active than that of gold. Therefore, higher activity of Ag/TNW than that of Au/TNW might confirm that silver was more active as the plasmonic sensitizer than gold. This is quite reasonable considering both possible mechanisms (electron and energy transfer), i.e., for energy transfer the similar energies should be transferred, and for electron transfer the lower work function of silver than that of gold might allow its easier transfer above Schottky barrier. In this regard, the lower activity of Ag/OAP than that of Au/OAP was surprising, and could be only explained by the co-existence of Ag_2_O/TiO_2_ heterojunctions with probably lower vis activity than plasmonic excitation, as already suggested in previous reports [66,101].

In order to compare this study with previous findings, fifteen commercial titania samples, including ST01 and TIO10, were chosen for the discussion. The vis activity of gold-modified TNW, OAP and commercial titania samples (activity reported before [29]) are presented in Figure 9b. Previously, it was found that Au/OAP showed the highest activity among all samples, even those with much better photoabsorption properties (larger gold crystallite size means here also higher polydispersity, and thus much broader LSPR peak.). It was proposed that faceted NPs might allow fast electron transfer via shallow ETs, and thus the main shortcoming of plasmonic photocatalysis (fast “back” electron transfer to NM) could be hindered [70]. Interestingly, here it was found that also Au/TNW showed much higher activity than that by fine titania samples with similar photoabsorption properties (size of gold NPs), suggesting that controlled morphology of support might enhance vis activity, probably by fast “hot” electron transfer via 1D nanostructure. Consequently, it was proposed that further improvement of morphology/properties (e.g., better crystallinity of TNW) should result in significant enhancement of vis response. For example, photocatalysts with special/complex morphology have been proposed to avoid this “back” electron transfer, such as deposition of gold NPs on the titania “superstructure” allowed an electron transfer from the basal surfaces to the edges of the plate-like mesocrystals through the TiO_2_ nanocrystal networks [78].

## 4. Summary and Conclusions

It has been found that noble metal (Au, Ag or Pt)-modification of TNW and OAP improves significantly their photocatalytic performance. The level of enhancement correlated with the type of photodeposited metal (Au, Ag or Pt), morphology (octahedron or wire) and reaction system (UV/vis or vis). Hydrogen liberation correlated with work function of metals, and thus platinum-modified samples showed the highest reaction rates. Photocatalytic activities of bare and modified OAP samples were much higher than those of TNW sample (except Ag-modified samples under vis irradiation), probably due to much higher crystallinity. Noble metals showed different influences on the activity, related to semiconductor support, i.e., gold-modified TNW and platinum-modified OAP possessed the highest activity for acetic acid decomposition, whereas silver- and gold-modified respective samples were the most active under vis irradiation. It is proposed that the form of noble metal (metallic vs. oxidized) as well as the morphology (well-organized vs. uncontrolled) had a critical effect on the overall photocatalytic performance. TRMC analysis confirmed that the rate of signal decay, i.e., the rate of electron scavenging (a decrease in charge carriers’ recombination), was the key factor for photocatalytic activity of NM-modified samples under UV irradiation. Whereas, under vis irradiation (plasmonic photocatalysis), the oxidation form of metal (zero-valent preferable), photoabsorption properties (broad LSPR), kind of metal (silver) and hindering of “back” electrons transfer to NM (by controlled morphology and high crystallinity) were a key factor for effective vis response.

## Figures and Tables

**Table 1:** Surface composition of modified samples determined by X-ray photoelectron spectroscopy (XPS) analysis of oxygen, titanium, carbon and metals for various samples.

Samples	Content (at%)						Ratio		Metal Content (wt%)		
	Ti	O	C	Au	Ag	Pt	O/Ti	C/Ti	Au	Ag	Pt
OAP *	5.3	27.8	66.9	-	-	-	5.2	12.6	-	-	-
Au/OAP *	9.5	31.6	58.7	0.17	-	-	3.3	6.2	4.4	-	-
Ag/OAP *	19.5	40.5	39.0	-	0.97	-	2.1	2.0	-	6.7	-
Pt/OAP	8.7	24.4	66.6	-	-	0.37	2.8	7.7	-	-	10.4
TNW	26.7	51.2	22.1	-	-	-	1.9	0.8	-	-	-
Au/TNW	9.9	31.4	58.6	0.09	-	-	3.2	5.9	2.2	-	-
Ag/TNW	16.6	39.2	43.8	-	0.44	-	2.4	2.6	-	3.6	-
Pt/TNW	17.6	40.2	41.9	-	-	0.29	2.3	2.4	-	-	4.0

* data published previously [66].

**Table 2:** Fraction of oxidation states of Au, Ag and Pt from deconvolution of XPS peaks of Au 4f_7/2_, Ag 3d_5/2_ and Pt 4f_7/2_.

Samples	Valent State (%)			Valent State (%)		Valent State (%)	
	Au^(δ+)^	Au^(0)^	Au^(δ−)^	Ag^+^	Ag^(0)^	Pt^(δ+)^	Pt^(0)^
Au/OAP *	4.7	93.2	2.1	-	-	-	-
Ag/OAP *	-	-	-	93.7	6.3	-	-
Pt/OAP	-	-	-	-	-	33.1	66.9
Au/TNW	2.9	90.6	6.5	-	-	-	-
Ag/TNW	-	-	-	94.3	5.7	-	-
Pt/TNW	-	-	-	-	-	24.4	75.6

* data published previously [66].

**Table 3:** Fraction of oxidation states of Ti, O and C from deconvolution of XPS peaks of Ti 2p3/2, O 1s and C 1s.

Samples	Ti 2p_3/2_ (%)		O 1s (%)			C 1s (%)		
	Ti^4+^	Ti^3+^	TiO_2_/K_2_Ti_8_O_17_	Ti–OH ^a^	Ti–OH ^b^	C–C	C–OH	C=O
OAP *	99.5	0.5	29.6	41.9	28.5	68.2	20.7	11.1
Au/OAP *	97.5	2.5	46.9	34.5	18.6	73.5	16.8	9.7
Ag/OAP *	97.3	2.7	70.3	25.2	4.5	82.5	9.2	8.3
Pt/OAP	99.1	0.9	53.6	35.9	10.4	89.8	4.8	5.4
TNW	96.3	3.7	78.4	20.7	0.9	96.2	3.8	0
Au/TNW	95.3	4.7	45.5	31.7	22.8	66.4	25.0	8.5
Ag/TNW	96.1	3.9	66.5	27.2	6.2	83.5	8.6	7.9
Pt/TNW	97.1	2.9	65.5	20.9	13.6	55.9	35.8	8.3

Ti–OH ^a^: Ti–(OH)–Ti/Ti_2_O_3_/C=O, Ti–OH ^b^: Ti–OH/C–OH; * data published previously [66].

## Supplementary Materials

The following are available online at https://www.mdpi.com/2079-4991/9/10/1447/s1, Figure S1: Diffuse reflectance spectra of Au/TNW for different gold content (0.01 to 5 wt%) with BaSO_4_ and bare TNW as reference; Figure S2: The photographs of the gold-modified TNW samples with different gold contents (from 0.01 wt% to 5 wt%); Figure S3: Influence of gold amount (0.01 to 5 wt%) on the TNW photocatalytic activity of H_2_ generation under UV/vis irradiation and acetone generation under vis irradiation; Figure S4: Diffuse reflectance spectra of TNW modified with different metals (Au, Ag and Pt); Figure S5: Diffuse reflectance spectra of OAP modified with different metals (Au, Ag and Pt); Figure S6: Photocatalytic activity for methanol dehydrogenation on bare and metal-modified TNW and OAP; Figure S7: Photocatalytic activity for decomposition of acetic acid on bare and NM-modified TNW and OAP; Figure S8: Comparison of photocatalytic activity for methanol dehydrogenation on bare and NM-modified TNW and OAP; Figure S9: Comparison of photocatalytic activity for decomposition of acetic acid on bare and NM-modified TNW and OAP; Figure S10: Hydrogen evolution during NM photodeposition on commercial titania samples (FP-6, ST01 and TIO10); Table S1: Structural properties of photocatalysts; Table S2: Properties of NMs’ deposits and photoabsorption properties of NM-modified OAP samples.
