# Supplementary material for: Influence of Semiconductor Morphology on Photocatalytic Activity of Plasmonic Photocatalysts: Titanate Nanowires and Octahedral Anatase Nanoparticles
Source: Nanomaterials (Basel). 2019 Oct 11;9(10):1447. doi: 10.3390/nano9101447 (PMC6835321; doi:10.3390/nano9101447)
Supplement: Supplementary file 1 [file nanomaterials-09-01447-s001.pdf]

Electronic supplementary information for

Influence of Semiconductor Morphology on Photocatalytic Activity of Plasmonic Photocatalysts: Titanate Nanowires and Octahedral Anatase Nanoparticles

Zhishun Wei [1,2,*], Maya Endo-Kimura [2], Kunlei Wang [2], Christophe Colbeau-Justin [3] and Ewa Kowalska [2,*]

1 Hubei Provincial Key Laboratory of Green Materials for Light Industry, Hubei University of Technology, Wuhan 430068, China
2 Institute for Catalysis (ICAT), Hokkaido University, N21 W10, Sapporo 001-0021, Japan; m_endo@cat.hokudai.ac.jp (M.E.-K.); kunlei@cat.hokudai.ac.jp (K.W.)
3 Laboratory of Physical Chemistry, UMR 8000, University of Paris-Saclay, 91190 Saint-Aubin, France; christophe.colbeau-justin@u-psud.fr
* Correspondence: wei@cat.hokudai.ac.jp (Z.W.); kowalska@cat.hokudia.ac.jp (E.K.)

Table S1. Structural properties of photocatalysts

Name	Main crystalline form[a]	Crystallite size[a]/nm	BET[b]/m^2 g^{-1}	Morphology (%)
OAP	TiO_2 (anatase)	17	124	octahedral
TNW	$K_2Ti_8O_{17}$	1.1	360	wire-like
FP-6	TiO_2 (anatase)	15	97	particle
ST01	TiO_2 (anatase)	8	298	particle
TIO10	TiO_2 (anatase)	15	100	particle

[a]Determined by XRD for anatase (OAP, FP-6, ST01 and TiO10) and $K_2Ti_8O_{17}$ (TNW) forms. [b]Specific surface area estimated by Brunauer, Emmett and Teller method.

Table S2. Properties of NMs' deposits and photoabsorption properties of NM-modified OAP samples

Titania	NM	Sample color	λ_{max}[a] (nm)	Predominant crystalline form[b]	Crystal size of zero-valent metal[b] /nm	Crystal size of metal oxide[b] /nm
OAP	Au	violet	544	Au	5.7	-
OAP	Ag	brown-violet	415	Ag_2O	7.5	14.6 (Ag_2O)
OAP	Pt	grey	405	Pt	5.4	-

[a]maximum extinction from DRS. [b]estimated from XRD.

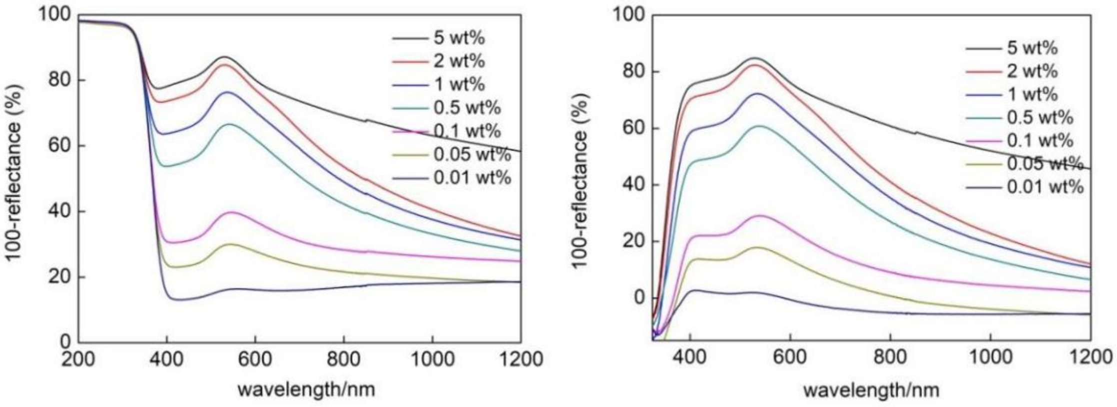

Figure S1. Diffuse reflectance spectra of Au/TNW for different gold content (0.01 to 5 wt%) with BaSO4 (left) and bare TNW (right) as reference, respectively.

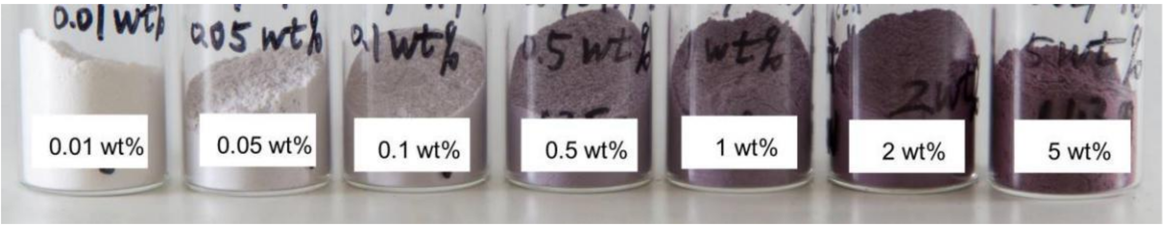

Figure S2. The photographs of the gold-modified TNW samples with different gold contents (from 0.01 wt% to 5 wt%; from the left).

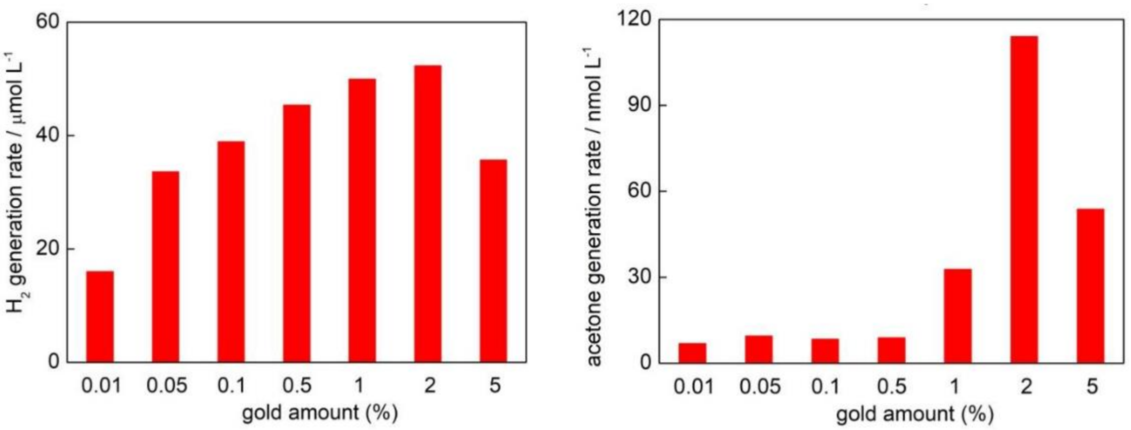

Figure S3. Influence of gold amount (0.01 to 5 wt%) on the TNW photocatalytic activity of H2 generation under UV/vis irradiation (left) and acetone generation under vis irradiation (right).

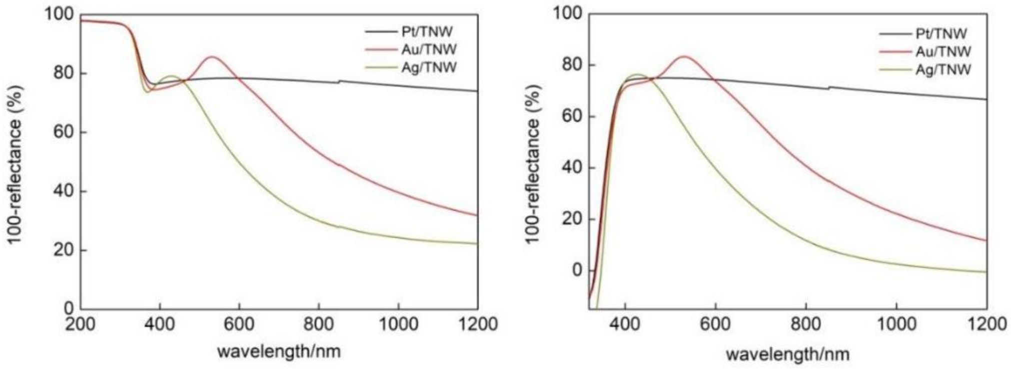

Figure S4. Diffuse reflectance spectra of TNW modified with different metals (Au, Ag and Pt). Spectra taken with BaSO4 (left) and bare TNW (right) as baseline.

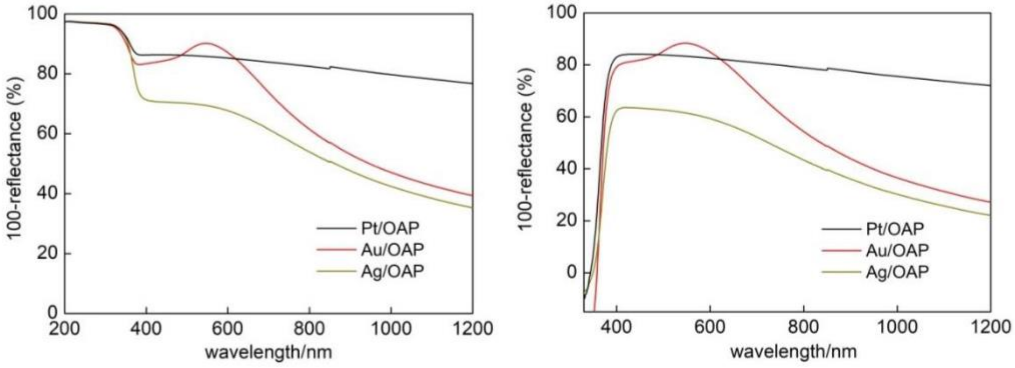

Figure S5. Diffuse reflectance spectra of OAP modified with different metals (Au, Ag and Pt). Spectra taken with BaSO4 (left) and bare TNW (right) as baseline.

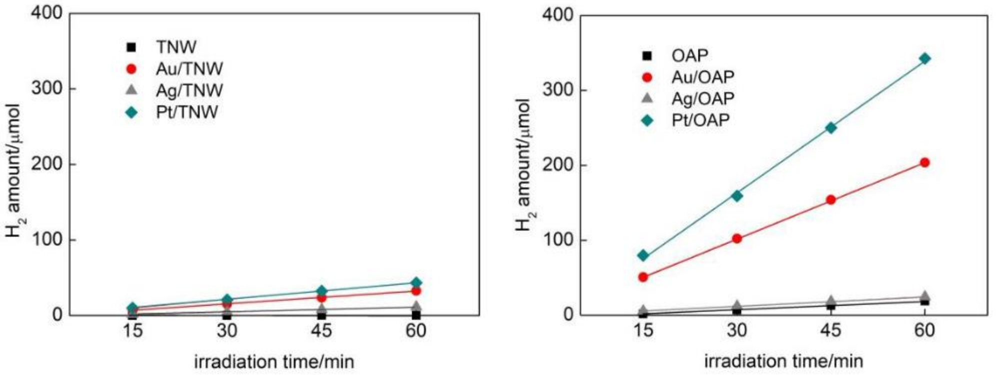

Figure S6. Photocatalytic activity for methanol dehydrogenation on bare and metal-modified TNW (left) and OAP (right).

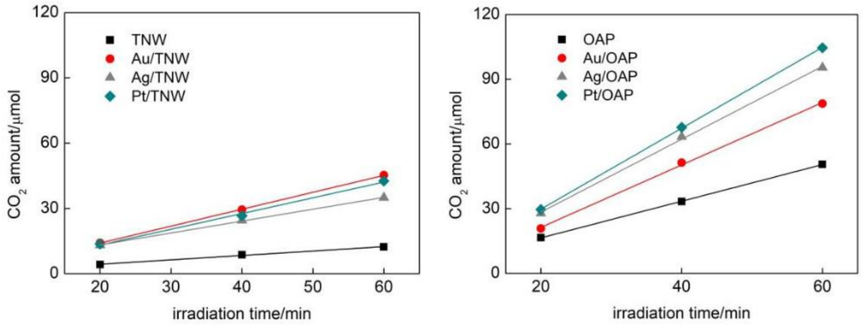

Figure S7. Photocatalytic activity for decomposition of acetic acid on bare and NM-modified TNW (left) and OAP (right).

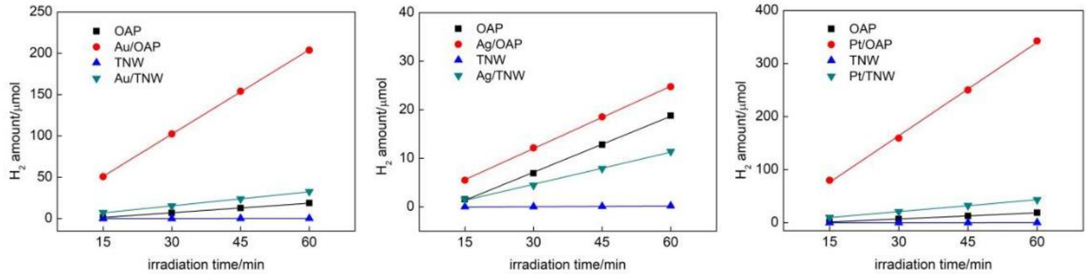

Figure S8. Comparison of photocatalytic activity for methanol dehydrogenation on bare and NM-modified TNW and OAP: (left) Au, (center) Ag, (right) Pt.

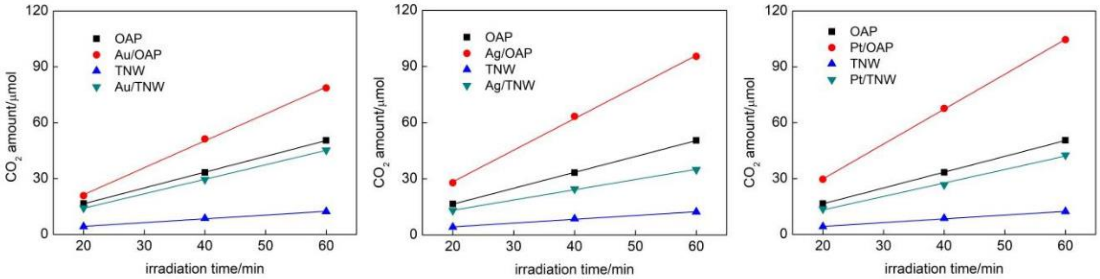

Figure S9. Comparison of photocatalytic activity for decomposition of acetic acid on bare and NM-modified TNW and OAP: (left) Au, (center) Ag, (right) Pt.

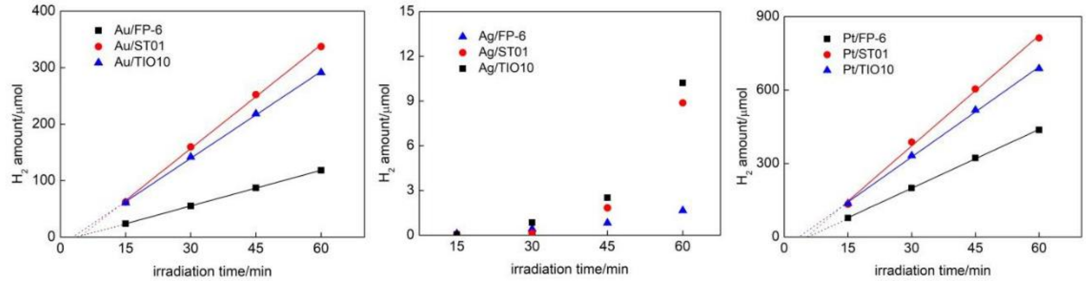

Figure S10. Hydrogen evolution during NM photodeposition on: (left) Au on FP-6, ST01 and TIO10, (center) Ag on FP-6, ST01 and TIO10, (right) Pt on FP-6, ST01 and TIO10.
